# How an Insect Converts Time Into Space: Temporal Niches Aid Coexistence via Modifying the Amount of Habitat Available for Reproduction

**DOI:** 10.1111/ele.70139

**Published:** 2025-05-30

**Authors:** Runa K. Ekrem, Alexander Jacobsen, Hanna Kokko, Tobias S. Kaiser

**Affiliations:** ^1^ Department of Evolutionary Biology and Environmental Studies University of Zurich Zurich Switzerland; ^2^ Max Planck Research Group Biological Clocks Max Planck Institute for Evolutionary Biology Plön Germany; ^3^ iomE & IQCB University of Mainz Mainz Germany

**Keywords:** biological clock, circalunar rhythm, *Clunio marinus*, competitive exclusion, intertidal zone, reproductive timing, spatiotemporal dynamics

## Abstract

Temporal niches do not automatically promote coexistence. We combine field data on the marine midge *Clunio marinus* with a model. In Roscoff (Brittany, France) sympatric *C. marinus* timing strains emerge at full moon (FM strain) or just before new moon (NM strain). We show that NM individuals reproduce and lay eggs when the water level is higher than during FM strain reproduction, and that this shift partially segregates larvae according to elevation. Modelling the underlying dynamics shows that the causality from temporal to spatial niches is crucial for coexistence: for hypothetical strains which differ in the temporal niche used for reproduction so that they use equivalently low water levels for egg‐laying, the dynamics show priority effects, not coexistence. While general theory on temporal niches is rather complex, we highlight the understudied possibility that timing traits cause differences in space use, and coexistence is unproblematic as it results from spatial niches.

## Graphical abstract

Temporal niches do not automatically promote coexistence. Combining field data and modelling, we show that in the marine midge *Clunio marinus* , sympatric timing strains, which reproduce during full moon or just before new moon respectively, can coexist because their different reproductive timing entails spatial separation of the larvae.

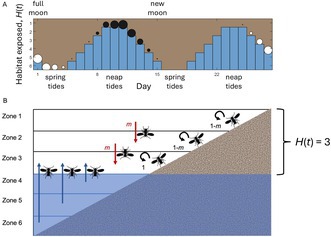

## 1 Introduction

Sympatric coexistence is a classic ecological problem (Gause 1934; Halperin 2025). Its enigmatic nature arises from the theoretical prediction that even small fitness differences should cause competitive exclusion of the less fit species (Armstrong and McGehee 1980; Chesson 2000), if one assumes that the species compete for the exact same resource. Nevertheless, in nature, there are sometimes very large numbers of species coexisting without apparently relying on distinctly different resources, the most famous example being the paradox of the plankton (Hutchinson 1961). This means that the first task for an ecologist looking for an explanation behind sympatric coexistence is to examine niche differences (Gause 1934).

Ever since Gause (1934), the field has struggled to match observed patterns to theoretical predictions (Halperin 2025, offers an interesting historical look into Gause's own work). The task is comparatively simple if the relevant axis of a resource is, for example, food or an ability to partition the spatial mosaic of resources across two (or more) competitors: in these cases, an increase in the number of competitors is more strongly felt by the conspecific individuals, and the intensification of intraspecific competition is theoretically known to promote coexistence (Chesson 2000; though questions still remain regarding shapes of density dependence that apply in nature and whether the most realistic choices explain coexistence, Aguadé‐Gorgorió et al. 2025).

Not all situations, however, are simple. We focus here on the intertwined role of temporal niches and space use. On their own (without a space use component which we turn to below), temporal niches might not yield coexistence as easily as one might intuitively assume. Consider a simple thought experiment where species A consumes a resource at time point *t* = 1 and species B arrives later, at *t* = 2, attempting to consume the same resource; A is at *t* = 2 dormant or away in some sense or another. In a coexistence‐promoting scenario, A depleted the resource, which then regrew (or new resource items arrived via e.g., water flow) to a level that allows B to reproduce. In this case, increasing A's density harms A's success more than B's, and temporal niches succeeded in concentrating competition to within a species.

Alternatively, however, the resource might recover much more slowly. If little has regrown after an overabundant A has consumed it, B simply arrives to find nothing to consume; A is then likely to win via a priority effect (Zou and Rudolf 2023). Thus, although temporal niches are in principle able to create conditions for coexistence (Meszéna et al. 2006; Gao et al. 2020), the task in each case is to examine if the conditions are fulfilled where regulating factors and species' responses to them become species‐specific (Barabás et al. 2012). Recent work has pointed out how often real‐life temporal niches fail to provide the necessary mechanisms: Stump and Vasseur (2023) emphasise just how many assumptions have to be met before they truly can make the theoretical basis of coexistence sound, and show that many real‐case scenarios fail to satisfy the relevant assumptions.

The situation is more complex still when competitors can change their location (dispersal at any life stage). It is possible that dispersal concentrates competition to mainly occur between conspecifics (Kareiva 1990; Amarasekare 2003), but this is not guaranteed. Thus, under intertwined space and time use, the task remains the same: to quote Meszéna et al. (2006), “spatio‐temporal heterogeneity does not weaken the necessity of having regulating/stabilizing mechanisms to equalize the long‐term growth rates”.

Here we provide a real‐life example that seeks to understand how the above considerations play out when competitors ‘A’ and ‘B’ are capital—rather than income—breeders (Stephens et al. 2009), which implies that larvae (the life stage that builds the ‘capital’) may compete even if adults that produced them used distinct temporal niches for reproduction (Ekrem et al. 2025).

We study the marine midge *Clunio marinus* (Haliday 1855), which has a life history tightly linked to tidal and, consequently, lunar cycles (Neumann 1986; Kaiser 2014). Larvae develop underwater for 6–14 weeks. The short‐lived adults (1–2 h) emerge and reproduce during low tide on specific days of the lunar cycle, when the spring tides expose the larval habitat for oviposition. There are different timing strains, e.g., some reproduce only during new moon (NM strains), others only during full moon (FM strains). FM and NM strains were found to coexist in Roscoff (Brittany, France; Kaiser et al. 2021). Adults do not feed, thus the important aspect for explaining coexistence is to understand regulating factors that occur among the larvae. Since larval development is far longer than the 2 weeks that separate adult emergence at the new and the full moon, there is potential for larval strains to compete even if the adults that produced them did not coexist in time.

**FIGURE 1:**
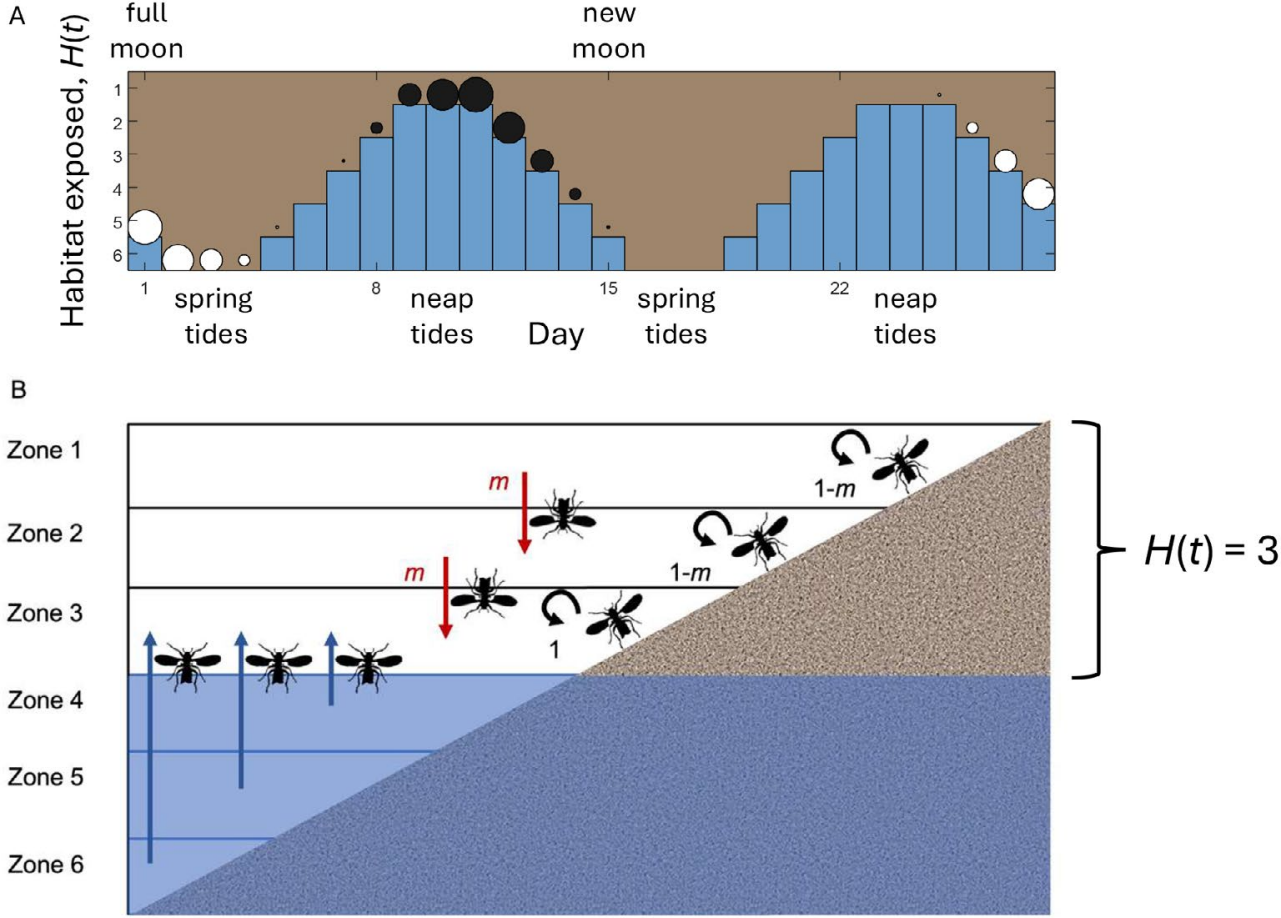
(a) Overview of the physical environment over 1 month, modelled as 28 days in the simulation. Spring tides expose the largest amount of habitat *H*(*t*), neap tides only expose one zone. Spring tides occur soon after the full moon (Day 1) and new moon (Day 15). Two competing strains are depicted, with emergence peaking at full moon (white circles) and 4 days before the new moon (black circles), corresponding to the FM and NM strains; the model also considers any other timing choices. The areas of the circles visualise the daily abundance of emerging adults, with this example assuming *v* = *q* = 5. Note that the two strains do not overlap in time, while they overlap, to a small degree, in the zones they use. (b) Overview of migration routes during low tide, exemplified for a day with *H* (*t*) = 3 (Days 7, 13, 21 and 27 have this value of *H*(*t*)). All individuals emerging in submerged zones migrate upwards to the closest exposed zone where *z* = *H*(*t*) (blue arrows). Above the lowest exposed zone, a proportion *m* migrates one zone down (red arrows), while nonmigrants (proportion 1–*m*) stay to oviposit in their natal zone (black arrows).

Since *C. marinus* adults usually oviposit near the water line (see Data S1), the location of oviposition and consequentially of the larvae is expected to be sensitive to the water levels during low tides. Low‐tide water levels vary throughout the lunar cycle (Figure 1). The new moon and the full moon produce the lowest water levels (*spring tides*), while the water stays relatively high during low tide when the moon is at first or third quarters (*neap tides*).

Here, we examine whether varying water levels create the necessary conditions for coexistence of strains, i.e., if there is a causal relation between temporal and spatial niches, so that timing differences in reproduction translate into space use differences in the larvae (Figure 1a). We document the novel finding that the sympatric FM and NM strains of *C. marinus* not only reproduce during different lunar phases in the field but also differ subtly in whether they align precisely with the spring tides (*matched emergence*; FM strain) or use a different set of days near the spring tides (*time‐shifted emergence*; NM strain). By genotyping larvae along a depth gradient, we then show that these temporal differences yield spatial separation of the larvae. This is because the waterline, where *C. marinus* oviposits, is differently located during reproduction of the two strains. The FM strain's matched emergence results in oviposition at lowest elevations, while the NM strain's time‐shifted emergence results in ovipositing at higher levels. We also model the consequences of this finding, confirming that coexistence is possible even if the spatial segregation of larvae is not complete; but coexistence fails if temporal differences are too mild to yield sufficient spatial segregation.

## 2 Methods

### 2.1 Study Species: Natural History and Location

The intertidal midge *Clunio marinus* is a capital breeder, in which emergence, mating, oviposition, and death all take place within an adult lifespan of a few hours during one low tide. Winged males scan the water surface for emerging wingless females, with whom they copulate, and thereafter position the female on exposed algae to oviposit. The long‐lived larvae (6–14 weeks) live in the lower intertidal zone, almost permanently submerged. It is therefore crucial that adult emergence, and thus mating and oviposition, are synchronised to the lowest tides (spring tide low tides) by a combination of endogenous circalunar and circadian clocks (Neumann 1966; Kaiser 2014). As spring tides occur around full moon and new moon, and as on these days there are two low tides (12.4‐h tidal cycle), there are four possible timing niches for reproduction, which are occupied by different timing strains of *C. marinus* (Kaiser et al. 2021). More details on *C. marinus'* life cycle are found in the Data S1.

Two timing strains co‐inhabit the intertidal of Roscoff (Brittany, France). Laboratory experiments predict that the FM strain emerges during the full moon spring tide, while the NM strain emerges in a time‐shifted manner a few days before the new moon spring tide (Kaiser et al. 2021). Both strains emerge during the evening low tide.

We conducted field work in Roscoff for 7 weeks from mid‐March to early May 2019. The field site, just below Roscoff Marine Station (SBR), features a shallow elevation gradient with small rock pools. The habitat consists of rocks with sand in between. The difference between high and low tide ranges from 2–5 m (neap tides) to 6–9 m (spring tides).

### 2.2 Adult Reproductive Timing in the Field

**FIGURE 2:**
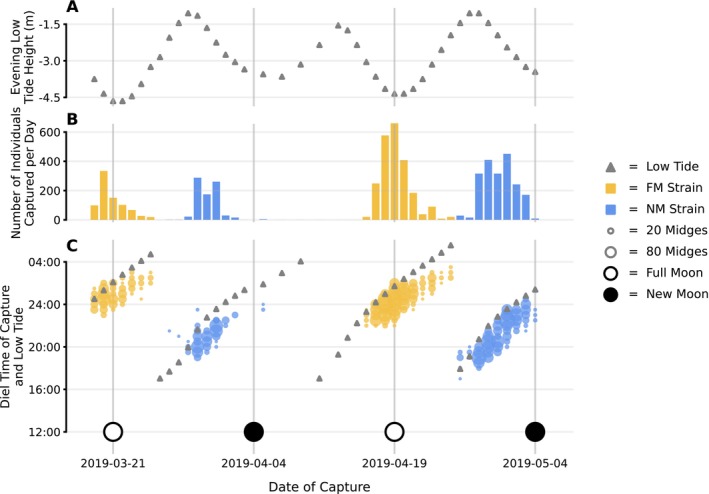
(a) Low tide water levels for evening low tides throughout our field period. (b) The number of individuals caught in the field each sampling day (total *N* = 5810). (c) The number of field‐caught individuals (represented by the size of the circle) per 30‐min interval and sampling day. Grey triangles indicate the time of the evening low tide for each day, and the phase of the moon is indicated in open and closed circles for full moon and new moon, respectively.

To include all natural cues that affect reproductive timing and space use, we tracked the temporally fluctuating abundance of *C. marinus* adults in Roscoff for the first time in the field. In late afternoons and evenings, we collected adult males in 30‐min intervals into 50 mL ethanol‐filled containers. Except for the first few days, we also recorded 30‐min intervals during which no adults were observed. All captured individuals were counted. The sampling strategy was adapted according to adult density and as the season progressed (see Data S1). This makes statistical analysis difficult. We thus simply present raw data of sampled individuals, which fortunately indicate very strong periodic fluctuations in line with *C. marinus'* known natural history (Figure 2).

### 2.3 Larvae Collection, Genotyping and Analysis

To assess the larval depth distribution of the two strains, we collected larvae during daytime low tides, scraping algae/sand substrates off rocks. We searched for larvae by mixing the substrate with sea water on white plastic plates. We inferred the elevation of collection sites (GPS‐recorded), based on a topographic map provided by Laurent Lévèque (Roscoff Marine Station, SBR). Additionally, a map of sample locations was generated in QGIS with bathymetric data from the Litto3D database (Louvart and Grateau 2005; QGIS.org 2021), combined with tidal data from the SBR.

Previous whole‐genome sequencing of 24 individuals of each strain revealed no diagnostic markers for FM vs. NM strains (pairwise F_ST_; Briševac et al. 2023). To assign larvae to one of the two strains, we therefore designed a multi‐locus genotyping assay based on the 11 most divergent loci in the published F_ST_ screen.

We used 94 fourth‐instar larvae from 49 sampling sites, extracting DNA from entire larvae following Reineke et al. (1998). We amplified target loci via two multiplexed PCR reactions (QIAGEN Multiplex PCR protocol; primer sequences in Table S1). We mixed reaction products in equal quantities for each larva and cleaned the products with ExoSAP‐IT (Applied Biosystems). Libraries were prepared for sequencing on an Illumina MiSeq platform according to standard protocols. In order to attain sufficient coverage for all loci, we concatenated reads over three separate sequencing runs (raw reads on EDMOND repository; https://doi.org/10.17617/3.LNP6DC).

We aligned sequence reads to the *C. marinus* genome (Kaiser et al. 2016; updated version CLUMA2.0 on EDMOND repository; https://doi.org/10.17617/3.42NMN2) using BWA mem (Li 2013) and filtered for mapping quality > 30. We truncated FASTQ files to avoid any locus exceeding 60× depth, as high sequencing depth artificially increased heterozygosity in the genotype calling. After sorting and indexing reads, we called single nucleotide polymorphisms (SNPs) using GATK's haplotype calling tools (Poplin et al. 2017).

One SNP per locus was selected for genotyping, based on criteria from the published F_ST_ screen. We removed all SNPs with F_ST_ < 0.3 and then selected SNPs with a major allele frequency > 0.8 in one strain and a minor allele frequency < 0.4 in the other strain. This resulted in 8 loci with scoreable SNPs. For each locus, we chose the SNP with the fewest missing genotypes across all samples for scoring. A SNP was scored as 1 if it contained two copies of the typical FM allele, 0 if it contained two copies of the typical NM allele, and 0.5 if it was heterozygous. Each individual's genotype score was defined as the average over all 8 loci.

This approach failed for the most divergent locus between FM and NM strains (*period*; Briševac et al. 2023), which was therefore scored based on a known insertion–deletion polymorphism (see Data S1).

As a test for the assay, we genotyped field‐caught adults with known emergence time in the same way (Figure S3).

We used the lme4 package in R (Bates et al. 2015; R Core Team 2021) to build a generalised linear mixed effects model for the relationship between the sampling depth (predictor variable) and the larval genotype score (response variable). We employed the logit link function as the response variable values ranged from 0 to 1. Sampling site was used as a random effect.

### 2.4 Modelling the Dynamics

#### 2.4.1 Aim and Overview

Our model scrutinises the consequences of timing differences for coexistence, when temporal niches of emergence impact space use during oviposition due to changing water levels. We approximate the real 29.53‐day lunar cycle as 2 × 14 days; we assume the first full moon occurs on Day 1 and the first new moon on Day 15 (Figure 1a).

The model's overarching aim is to investigate whether two strains that differ in their temporal niches can coexist (Figure 1a shows an example with timing peaks corresponding to real‐life FM and NM strains). It appears intuitive that the timing differences are stark enough in Figure 1a to cause larvae mainly to compete with others of the same strain, yet intuition needs to be checked against tracking the dynamics, which we follow for 100 years. In addition to real‐life timing differences (Figure 1a), we explore systematically whether all possible temporal niche differences, or only a subset of them, yield coexistence. We do this by computing the pairwise invasion for every combination (peak at 1, 2, …, 28) of invader and resident strain, recoding time‐averaged proportions after invasion succeeded to yield coexistence (over 100 years), succeeded in outcompeting the resident, or failed.

Invaders are assumed initially rare: each simulation run starts with the invader constituting 1% of the population and ends when the 28‐day mean of the resident strain frequency changes by less than 10^−5^, or if the maximum simulated time of 100 years was reached. 100 years defines a realistic range for ecological coexistence, but in practice, the frequencies stabilised (including the possibility that one strain went extinct) before this cap was reached.

Our simulation setup also tests the intuition that timing differences yield coexistence if, and only if, they cause sufficiently strong spatial segregation (such that in the absence of spatial segregation, the resident or the invading strain completely outcompetes the other). To quantify the degree of spatial separation between the larval populations of two strains, we use the ‘sexual segregation and aggregation statistics’ (SSAS) of Bonenfant et al. (2007). We label it SC (‘segregation coefficient’), as our context does not involve spatial separation of the sexes (see Data S1 for details of implementation). Its values range from 0, complete spatial overlap, to 1, complete segregation. Note that SC also becomes zero if there is only one strain left.

#### 2.4.2 The Habitat

We assume that adults are only present during evenings (matching our field data), and consequently track the amount of intertidal habitat exposed, *H*, during one low tide each day. Since males move females around during mating, we divide the beach into six vertical zones, which gives sufficient spatial resolution and allows us to model different beach profiles (see below), while not creating an unrealistically narrow zone in which one female is assumed to lay eggs. The amount of habitat available thus ranges from 1 to 6 (*H* = 1, 2, …, 6) along an elevation gradient (Figure 1a).

As the water recedes, habitat zone 1 becomes exposed first, followed by 2 and so on. But not all low tides expose all zones (Figure 1a depicts the lowest level that the water reaches on each day). We discretise the relevant sine wave by assuming that the spring tide exposes all six zones for 3 days (*H*(*t*) = 6 on days *t* = 2, 3, 4, i.e., shortly after the full moon, and 16, 17, 18, i.e., shortly after the new moon) while the neap tides only expose one zone (*H* = 1) for 3 days. The number of exposed zones changes by one zone/day on surrounding days. We use the subscript *z* to denote zones when assigning individuals to their locations; thus e.g., *z* = 3 describes a larva or an adult that is currently residing in zone 3.

#### 2.4.3 Strains and Their Timing Traits

We track the dynamics of two strains, resident (R) and invader (I), that differ in their timing traits; we also use S to denote ‘strain’ in equations that are identical in form but calculated separately for each strain. Strains are defined by the day of their emergence peaks. In real life, the FM strain peaks on Day 1 (full moon), while the NM strain peaks on day 11, i.e., 4 days before new moon (Figure 1a). We study their competition, but also all other potential peak days for the R and I strains.

We assume that emergence begins 4 days before the peak day and ends 4 days afterwards, with a Beta distribution that can be varied for how strongly emergence is centered around the peak (see Figure 1a for a fairly pronounced peak; Data S1 has equations and a list of all variables).

#### 2.4.4 Dispersal of Emerged Adults

We assume that adults disperse for two reasons, corresponding to two vertical directions (Figure 1b). *Upwards migration* occurs because emergence from submerged zones is permitted, but ovipositing in submerged zones is not possible. Thus, all females emerging within submerged zones (*z* > *H*(*t*)) move to the lowermost exposed zone (*z* = *H*(*t*)). *Downwards migration* is not obligatory, but may occur, reflecting *C. marinus'* tendency to oviposit close to the waterline (see Supplement on life history). Females emerging in high‐elevation zones may therefore be carried downwards by males. We define *m* as the proportion of individuals moving by one zone towards the water's edge (from *z* to *z* + 1), conditional on emergence occurring at locations where habitat at *z* + 1 is exposed (*H*(*t*) ≥ *z* + 1 must be true). We present results for *m* = 0.2, 0.5, and 1.0.

#### 2.4.5 Reproduction

We assume each female produces *C* = 50 daughters of the same strain as herself (see details on life history in Data S1) and thereafter dies. Eggs are laid in the zone where the female resides post‐dispersal.

#### 2.4.6 Larval Development and Density‐Dependent Mortality

The model tracks the number of female larvae that proceed through development stages *i* = 0, 1,…, 21. While we do not assume stage‐structured survival (but density‐dependence, see below), the model requires repeated probabilistic transitioning between stages (daily probability *r*) to create a realistic distribution for the time that it takes to complete larval development (see details on life history in Data S1). We use *r* = 0.45, which implies that in the absence of mortality, larval development takes on average 21/0.45 = 47 days, but since stage transitions are stochastic, the model avoids unrealistic synchrony and instead makes larvae ready to emerge over a time span roughly between 1 and 2 months (Figure S1). Individuals of the penultimate developmental stage (*i* = 20) do not use *r* but instead transition to the final stage (*i* = 21) 16 days before their upcoming strain‐specific emergence window (see Supplement on life history). All final‐stage survivors emerge in that window.

We assume population regulation at the larval stage since adults do not eat, and it is difficult to envisage a process that would make reproductive success decline with increasing adult density. Every larva is subject to daily density‐dependent mortality. All same‐zone larvae contribute to density (and thus the competitive environment), regardless of their strain. Survival on day *t* is

(1) $$s_{z}\left(t\right)=s_{\max}e^{-\alpha_{z}{\left(N_{Lz}\left(t\right)\right)}^{2}}$$

where *s*_max_ denotes survival under unlimited resources, $\alpha_{z}$ indicates the zone‐specific strength of density dependence, and *N*_L*z*_(*t*) denotes the number of larvae residing in zone *z* at time *t*. High $\alpha_{z}$ implies survival declines faster with larval population size. Note the square term: its role is to make the model include the biologically plausible assumption that survival does not begin declining appreciably until there are many competitors depleting the relevant resource (this is the 4th model in a classic paper, Bellows 1981, who found it to be among the best fitting models in various datasets).

In our baseline scenario, $\alpha_{z}$ has the same value across all *z*, but we also use variations in the value of $\alpha_{z}$ to model alternative assumptions regarding beach topography: narrow zones or less resources are modelled by assigning them a high value of $\alpha_{z}$ (Data S1).

## 3 Empirical Results

### 3.1 Strain‐Specific Emergence During Full Moon or Before New Moon

The first ever field data on the FM and NM strains' emergence show a bimodal distribution within each lunar cycle (Figure 2b,c), reflecting the known emergence of the laboratory strains (Kaiser et al. 2021). Between the emergence period of the FM strain and the following emergence period of the NM strain there was no day without emergence. However, this overlap between the temporal niches is superficial; on a diel temporal scale they are clearly distinct (Figure 2c). The expectation from laboratory results, that the strains differ in their alignment to spring tides, was confirmed in the field (Figure 2b). The emergence peaks of the FM strain matched full moon (*matched emergence*). The emergence peaks of the NM strain occurred 4–6 days before new moon (*time‐shifted emergence*). Consequently, the FM strain emerged during lower water levels than the NM strain (compare Figure 2a,b). Combined with a tendency to oviposit near the water's edge, this suggests that FM matched emergers oviposit at lower elevations than the NM time‐shifted emergers.

### 3.2 Circadian Emergence Time Is Modulated by the Tides

In the FM and NM laboratory strains, the strain‐specific circadian emergence time occurs at the same time of day regardless of the day in the lunar cycle (Figure S2b). In contrast, in the field, the diel abundance peak shifted each day in accordance with the low tide (Figure 2c and Figure S2a). Diel abundance was concentrated just before the low tide (Figure 2c). NM individuals generally emerged earlier within a day than FM individuals, corresponding to the earlier times of low tide on the days on which they emerge (Figure 2c). This modulation of diel emergence by the tide ensures that mating and oviposition happen exactly during low tide, which in turn underlines that the low tide level on the day of emergence critically determines oviposition sites.

### 3.3 Larvae of the FM and NM Strains Settle at Different Levels in the Intertidal Zone

**FIGURE 3:**
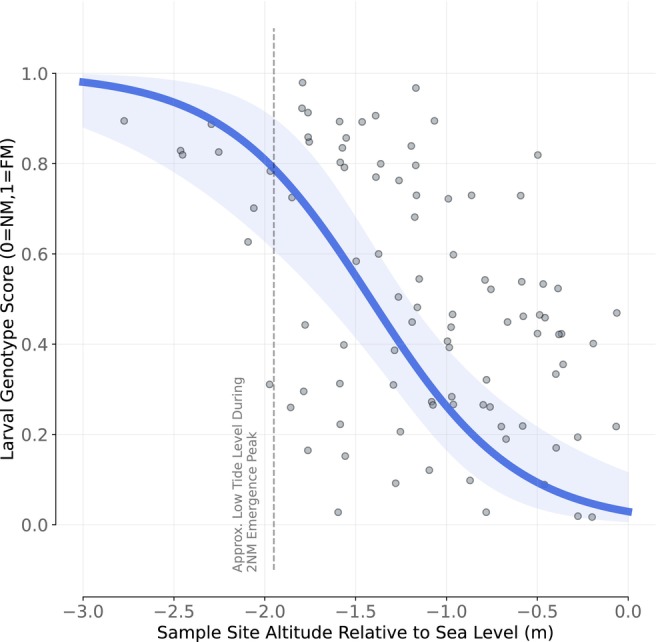
The distribution of larval genotype scores over the bathymetric sampling gradient. Each dot represents an individual larva, with slight jitter added to the plot to better visualise overlapping dots. The blue line is the fitted generalised linear mixed effects model, with the shaded region representing the 95% confidence interval. The dashed grey line indicates the approximate low tide level during the peak of emergence for the new moon strain. Intermediate sampling depths yield the full range of genotype scores, suggesting the two strains' spatial distributions overlap.

The observation that the FM strain emerges, mates and oviposits during lower low tides than the NM strain leads to the prediction that FM larvae develop lower in the intertidal zone than NM larvae. To identify the larvae of each strain, we developed a multi‐locus genotyping assay and confirmed its validity based on adults caught during full moon and new moon respectively (Figure S3). For the larvae, sampling depth indeed proved to be a significant predictor of their genotype (*p* < 0.001, *β* = −2.48), with genotypes shifting from typical NM towards typical FM with increasing sampling depth (Figure 3; Figures S4 and S5). Shallow locations harboured increased variability in genotype scores (from 0 to 0.5; NM; Figure S6), while deep locations were closer to ‘pure’ FM genotypes (Figure 3 and Figure S6). Intermediate elevations yielded larvae with the entire range of genotype scores, i.e., this part of the intertidal zone is occupied by both strains, as well as potential hybrids. The transition from high to low variability aligns with the low tide water level during the NM strain's emergence peak (Figure 3; Figures S2 and S6).

## 4 Model Results

**FIGURE 4:**
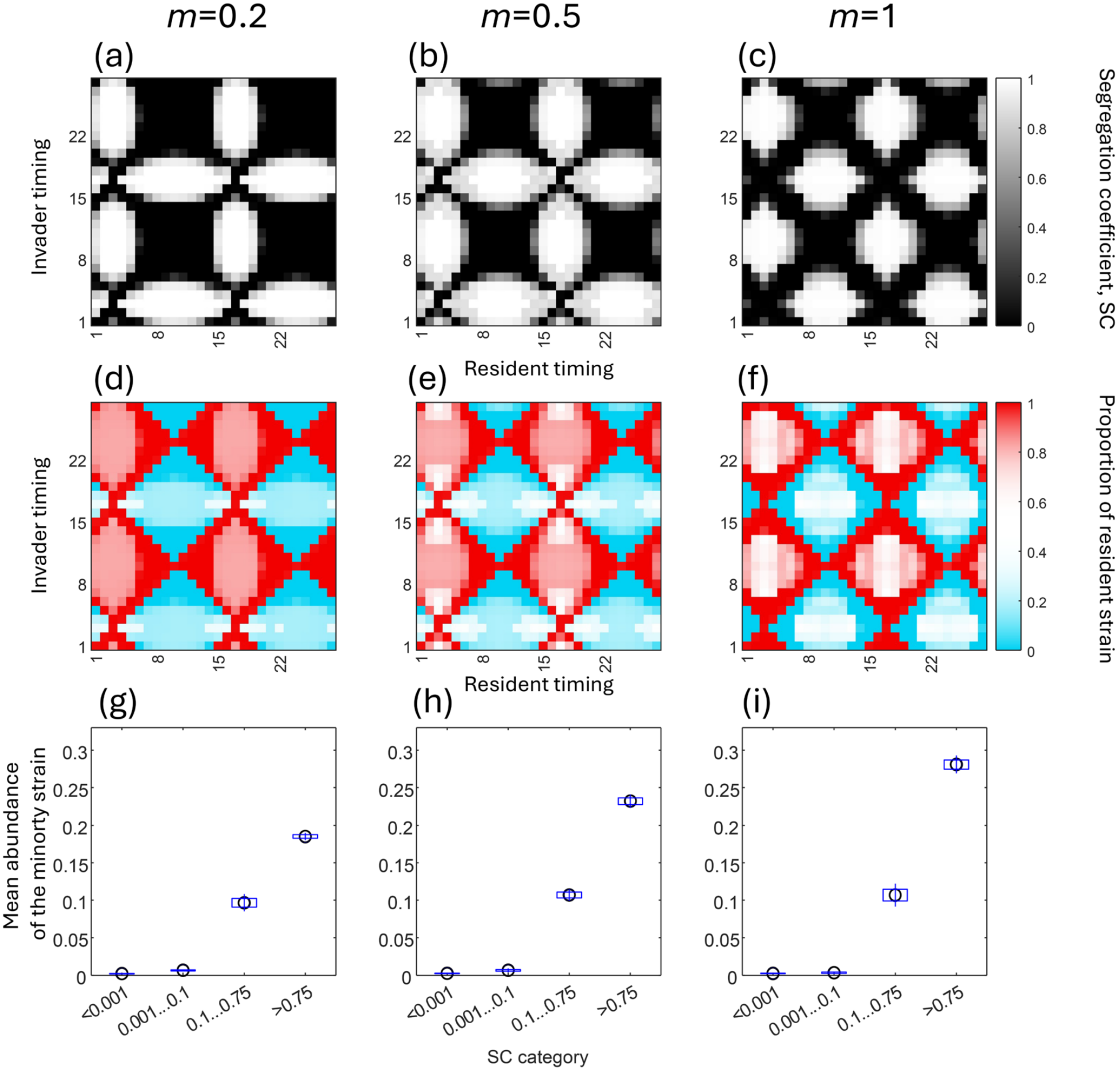
(a–c), Spatial segregation (SC; white: Complete segregation, black: Complete overlap). Values along the x and y axes give the peak of emergence for the resident and invader strains, respectively. Downwards migration rate is indicated for each column of panels (from left to right: *M* = 0.2, *m* = 0.5, *m* = 1). (d–f) Global strain frequencies (i.e., across all zones; red: 100% resident, blue: 100% invader) at the end of the simulation averaged over 28 days. x and y axes as in panels (a–c). (g–i) Mean ± SE and confidence limits for the values in (d–f), binned to four different SC categories. Note that extinction produces SC = 0, where causality is difficult to ascertain (did low SC cause extinction or did extinction force SC to zero?). Therefore, we plot separately the next category of low but non‐zero SC values, which strengthens our message that high abundance for the minority strain is only possible when SC is neither zero nor near zero. Constant parameters: *V* = *q* = 2, *α*_*z*_ = 10^−8^ for all *z*, *r* = 0.45.

The coexistence of the two strains depends crucially on their emergence schedules (Figure 4). While the details depend on the width of the temporal niches (Figure 4: broad, *v* = *q* = 2, Figure S7: narrow, *v* = *q* = 5) and on the scale of downwards migration (columns in Figure 4), the overall message remains consistent, which we explain using Figure 4a,d,g as an example.

If two strains emerge at the same time (diagonals in Figure 4a,d) or temporally separated by 2 weeks (e.g., one beginning on Day 1, another on Day 15), adults emerge at similar water levels, and larvae consequently experience strongly overlapping spatial niches (black in Figure 4a). If one strain is shifted such that the timing difference deviates strongly from being a multiple of 14 days (e.g., one emergence period begins on Day 1, another on Day 11), the larvae segregate spatially (white in Figure 4a).

The degree of spatial overlap translates into patterns of invasion success and coexistence (or lack thereof). Temporal niches that lead to a lack of spatial segregation (black in Figure 4a), correspond to blue or red regions in Figure 4d, where the invasion is wholly unsuccessful (red) or succeeds ‘too well’ for coexistence, i.e., displaces the resident (blue). Where temporal niches yield spatial segregation (white in Figure 4a), there is the possibility of coexistence (white in Figure 4d).

Thus, for coexistence in our system, it is essential that the differing temporal niches for reproduction cause spatial segregation of larvae. Not all temporal niche differences do this. In particular, non‐overlapping temporal reproduction that places larvae in the same depth zones, i.e., reproduction precisely 2 weeks apart, always yields a priority effect instead of coexistence (all diagonals are red if the invader is shifted by 14 days from the resident, Figure 4d–f and Figure S7–S9), regardless of the resident's emergence time. Figure 4g confirms the visual impression that white areas in Figure 4a (strong spatial segregation) covary with the location of the white areas in Figure 4d (coexistence of two strains). This holds true for other values of migration (rest of Figure 4) and widths of the temporal niches (Figure S7). The main effect of increasing downwards migration rates is that coexistence is more common (more white in Figure 4b,c,e,f).

The strains co‐occurring in Roscoff have their peaks scheduled asymmetrically. The NM strain peaks ~10 days after the FM strain (instead of a symmetry‐inducing 14 days). This combination of days yields spatial segregation and stable coexistence in every panel of Figure 4 (e.g., resident at Day 1, invader at Day 11). The intermediate‐to‐high spatial segregation values that the model predicts for *C. marinus* real emergence schedules imply that the two strains co‐occur at intermediate elevation zones. This is in line with our findings from the field (Figure 3).

### 4.1 Varying Density‐Dependence

The above results relied on the same strength of density dependence across all depth zones, which implicitly assumes that all zones offer equivalent resources. This assumption is simplistic, as e.g., the zones can be of unequal area due to topography or differ in the quality of resources given the strong change of biotic communities along depth gradients in the intertidal zone. To relax this assumption, we allowed the strength of density dependent mortality (*α*_*z*_) to differ between zones; as exemplary cases we linearly increased *α*_*z*_ with increasing depth (corresponding to a decrease in available area or resource quality) and the reverse.

Qualitatively, the results are similar to constant *α*_*z*_ (Figures S8–S9), with certain shifts in the results that are intuitively clear. If the habitat is larger or better at higher elevations, there are more individuals available to perform downward migration, which in turn facilitates coexistence, in line with the findings obtained when directly increasing downward migration (Figure 4a–f). Inversely, when there is larger or better habitat at lower elevations, upward migration is increased and coexistence is harder.

## 5 Discussion

A central task in understanding coexistence between species is to identify the regulating factors that inhibit the growth of a species (or in our case, strain) once it has become abundant. Temporal niches can do this under some circumstances, but not all. In the case of *C. marinus* , our work shows that this requires two strains to align differently to the spring tides. The two‐week difference between two spring tides alone (full moon vs. new moon) does not yield coexistence: should *C. marinus* strains divide the time axis with emergence every 2 weeks, coexistence would remain enigmatic, as the dynamics are driven by a clear priority effect (the invader fails; Ekrem et al. 2025 show that this result extends to scenarios with Allee effects and hybridization). Instead, our finding that one of the *C. marinus* strains (NM) emerges a few days early with respect to the spring tides (Figure 2) is key to understanding coexistence. The NM strain uses a higher part of the intertidal zone than its competitor, the FM strain, which matches its emergence with the lowest water levels (Figure 3). Our modelling then shows that these strain‐specific timing choices with respect to the lunar cycle separate the larvae into sufficiently different competitive pools.

Coexistence is much more strongly dependent on the exact timing choices than on how strongly we assume *C. marinus* to be attracted to the waterline once emerged. Emergence was observed just before low tide each day (Figure 2c). This suggests that the circadian clock sets a window during the day within which emergence can occur, and environmental stimuli (falling tide) then trigger emergence within that window. This implies that emergence occurs close to the descending waterline, helping to maintain the spatial separation of the strains without the need for long‐distance dispersal to oviposit near the waterline.

An interesting follow‐up question that our study does not yet address is whether the spatiotemporal separation is an evolved response to competition. Could the shift have evolved *because* it helps avoid competition coming from the presence of larvae of another strain? Pfennig and Pfennig (2009) define character displacement as trait evolution stemming from selection to lessen resource competition or reproductive interactions between species. While displacement is challenging to document unambiguously (Stuart and Losos 2013), it is interesting that timing traits have been included in the set of possible traits that can undergo character displacement. The phenology of flowering is a good example: while the timing of flowering is not commonly displaced in sympatry (compared with allopatry), evidence for displacement is stronger in those angiosperm species pairs that flower close in time (Park et al. 2022). *C. marinus* offers intriguing research avenues in this context, because the effects of timing traits on competition among larvae, which our study documents, differ from their effects on adult reproduction, and both competition and reproduction feature in the general theory for character displacement (Pfennig and Pfennig 2009). For competition, we have already shown that the shift helps to place larvae in locations with reduced inter‐strain competition, but we did not include heritable variation in the magnitude of the shift. For reproduction, allowing freely evolving emergence timing requires future models to be explicit about co‐emergence potentially producing hybrids. In laboratory crosses, hybrids have maladaptive intermediate timing phenotypes (Briševac et al. 2023), which suggests that a crucial requirement for reproductive character displacement is in place.

Our study has unique features due to the importance of moon phases, yet its lessons remain valid in (1) alternative contexts where temporal niches create spatial differences with the potential to enhance coexistence, and (2) more general contexts. Regarding (1), temporally varying waterline effects are important in river ecosystems with seasonal floods: they can have plants coexisting based on differences in dispersal phenology (Niiyama 1990; Fraaije et al. 2015), as seed dispersal during flooding establishes seedlings higher on floodbanks than dispersal at other times. In animals, coexistence is enhanced if temporal variation in reproduction matches specific water fluctuations, so that larvae produced at specific times travel in specific directions (Berkley et al. 2010; Aiken and Navarrete 2014).

Zooming out to the more general lessons (2), the outcomes of our study stress the importance to document that individuals of a relevant category really do settle in different parts of their habitat if temporal niches are argued to aid coexistence via translating into spatial separation. The ‘relevant category’ here refers to the fact that regulating and stabilising mechanisms (Meszéna et al. 2006) may be dependent on age or life history stage. In our particular case, the species is a capital breeder that gathers all resources required for breeding during larval development, and therefore temporal separation of adults, while necessary, is far from sufficient to yield coexistence.

Of course, the requirement for spatial separation is obsolete if it can be shown that the conditions for temporal separation producing species‐specific regulation are met in some other way (e.g., rapidly renewing resources and each consumer gathering its resources in a short time span before reproducing, as in our first example of the introduction, or more complex aspects of the storage effect, Stump and Vasseur 2023). The most general lesson from our study, therefore, is that it is important to appreciate that temporal niche differences do not always easily produce coexistence (Stump and Vasseur 2023), but they are not hopeless as an explanation either. In particular, whenever temporally varying habitat characteristics (including amount of habitat available) offer a potential causality from individuals' timing to their spatial locations, coexistence may find a very natural explanation in spatial segregation of competitors with alternative temporal niches.

## Author Contributions

H.K. and T.S.K. conceived and supervised the study. R.K.E. performed field work and mathematical modelling. A.J. performed genotyping and empirical data analysis. R.K.E. and A.J. wrote the first draft of the manuscript, and all authors contributed substantially to the drafts and gave final approval for publication.

### Peer Review

The peer review history for this article is available at https://www.webofscience.com/api/gateway/wos/peer‐review/10.1111/ele.70139.

## Supporting information


Data S1.

## Data Availability

All data and code used in this manuscript have been submitted to the EDMOND repository (https://doi.org/10.17617/3.LNP6DC).
