# Supplementary material for: How an Insect Converts Time Into Space: Temporal Niches Aid Coexistence via Modifying the Amount of Habitat Available for Reproduction
Source: Ecol Lett. 2025 May 30;28(6):e70139. doi: 10.1111/ele.70139 (PMC12123478; doi:10.1111/ele.70139)
Supplement: Supplementary file 1 — Data S1. [file ELE-28-0-s001.pdf]

Supplementary Material

Table of contents

Supplementary Text: *Clunio marinus* life history 2

Supplementary Methods: Adjustments in the sampling of adults 4

Supplementary Methods: Multilocus genotyping 5

Table S1 Primers for PCR amplification 5

Supplementary Methods: Modelling details 6

Table S2 List of variables and parameters 6

Table S3 The number of females ovipositing in each zone 8

Supplementary Figures 9

Figure S1 Larval development time distribution in the model 9

Figure S2 FM and NM strain emergence in the wild vs. the laboratory 10

Figure S3 Testing the multi-locus genotyping assay in field-caught adults 11

Figure S4 Larval sampling sites in Roscoff, France – tide levels 12

Figure S5 Larval sampling sites in Roscoff, France – bathymetry 13

Figure S6 Variation in genotype scores relative to depth in the intertidal 14

Figure S7 Model results for pointier emergence distribution 15

Figure S8 Model results for decreasing strength of density dependence with increasing depth 16

Figure S9 Model results for increasing strength of density dependence with increasing depth 17

Supplementary Text: *Clunio marinus* life history

Clunio marinus (Diptera: Chironomidae) inhabits the rocky shores of Europe's Atlantic coast, where it spends the majority of its life in the larval state, occupying patches of filiform macroalgae in the lower midlittoral zone (Neumann 1986). Generation times in the laboratory are typically between 6 to 14 weeks (Neumann 1986). While as larvae, individuals generally live within tubes constructed of silt and their own saliva and graze on microalgae and detritus (Neumann 1976). Reproduction is synchronized to the spring tide low tides (Neumann 1975, Kaiser 2014). An endogenous circalunar clock ensures that the late larval development and pupation are synchronized with the lunar cycle (Neumann 1975, Neumann & Spindler 1991). The lunar synchrony of the population is achieved by arresting larval development in the last instar during a specific waiting stage; after this developmental arrest has been broken, further larval development and the pupal stage take about 16-20 days until adult emergence (Neumann & Spindler 1991). A circadian clock times adult emergence to the low tide (Neumann 1975, Kaiser 2014). *C. marinus* is a capital breeder, in which emergence, mating, ovipositing and death all take place within an adult lifespan of typically less than 2 hours during one low tide (Neumann 1975). Winged males scan the water surface for emerging wingless females, with whom they copulate while hovering on the water surface, and thereafter position the female on emerging rocks and algae to oviposit (Neumann 1976, Neumann 1966). This generally limits oviposition to substrates that have just emerged, i.e. is oviposition usually happens very close to the current waterline. Eggs stick to the algae, which prevents the incoming tide from washing them off (Neumann 1976). A typical egg clutch contains on average 100 eggs, but can range between 70-150 (Neumann 1976, Neumann 1966). In our laboratory cultures we have not observed any deviation from a 50:50 sex ratio.

Spring tide low tides, the suitable tides for reproduction, occur around full moon and new moon. Additionally, there are two low tides a day, occurring 12h 25 min apart. This combined results in four suitable timing niches for reproduction in each lunar cycle (Kaiser et al., 2021). These timing niches are occupied by different timing strains of *C. marinus*. These timing strains emerge either around full moon only (FM strains), new moon only (NM strains) or both full moon and new moon (SL strains; for semilunar). Emergence is typically spread over about 5-7 days. Additionally, timing strains differ in whether they use the morning low tide (type 1) or the evening low tide (type 2). They always only use one of the two. The combination of daily timing (1 or 2) and lunar timing (FM, NM, SL) results in six possible timing strains: 1FM, 1NM, 1SL, 2FM, 2NM and 2SL. However, so far only 2FM, 2NM, 2SL and 1SL strains have been found (Kaiser et al., 2021). Timing strains also have finer adaptations to the local tidal regime, which differs for different places along the coastline (Kaiser et al. 2021, Neumann 1976). These local adaptations, as well as the differences between timing strains, are genetically controlled (Neumann 1967, Kaiser et al. 2011, Kaiser et al. 2016, Briševac et al. 2023).

The two sympatric strains we investigate in this study are from Roscoff (Brittany, France) and were therefore called Ros-2FM and Ros-2NM (Kaiser et al. 2021). For simplicity we call them only "FM strain" and "NM strain" in this study.

References

Briševac, D., Peralta, C. M., & Kaiser, T. S. (2023). An oligogenic architecture underlying ecological and reproductive divergence in sympatric populations. Elife, 12, e82825. https://doi.org/10.7554/eLife.82825

Kaiser, T. S. (2014). Local adaptations of circalunar and circadian clocks: The case of Clunio marinus. In H. Numata, B. Helm (Eds.) *Annual, Lunar, and Tidal Clocks: Patterns and Mechanisms of Nature's Enigmatic Rhythms* (pp. 121-141). Springer Japan. https://doi.org/10.1007/978-4-431-55261-1_7

Kaiser, T. S., von Haeseler, A., Tessmar-Raible, K., & Heckel, D. G. (2021). Timing strains of the marine insect *Clunio marinus* diverged and persist with gene flow. *Molecular Ecology*, *30*(5), Article 5. https://doi.org/10.1111/mec.15791

Kaiser, T. S., Neumann, D., & Heckel, D. G. (2011). Timing the tides: genetic control of diurnal and lunar emergence times is correlated in the marine midge Clunio marinus. BMC Genetics, 12, 1-12. https://doi.org/10.1186/1471-2156-12-49

Kaiser, T. S., Poehn, B., Szkiba, D., Preussner, M., Sedlazeck, F. J., Zrim, A., ... & Tessmar-Raible, K. (2016). The genomic basis of circadian and circalunar timing adaptations in a midge. Nature, 540(7631), 69-73. https://doi.org/10.1038/nature20151

Neumann, D. (1966). Die lunare und tägliche Schlüpfperiodik der Mücke *Clunio*. Zeitschrift für vergleichende Physiologie 53:1-61. https://doi.org/10.1007/BF00343045

Neumann, D. (1967). Genetische Adaptation der Schlüpfzeiten von Clunio-Populationen an verschiedene Gezeitenbedingungen. Helgoländer wissenschaftliche Meeresuntersuchungen, 15, 163-171.

Neumann, D. (1975). *Lunar and Tidal Rhythms in the Development and Reproduction of an Intertidal Organism*. In F.J. Vernberg (Ed.), *Physiological adaptation to the environment* (p. 451-463) Intext Educational Publishers, New York.

Neumann, D. (1976). Adaptations of Chironomids to Intertidal Environments. *Annual Review of Entomology*, *21*(1), 387–414. https://doi.org/10.1146/annurev.en.21.010176.002131

Neumann, D. (1986). Life Cycle Strategies of an Intertidal Midge Between Subtropic and Arctic Latitudes. In F. Taylor & R. Karban (Eds.), *The Evolution of Insect Life Cycles* (pp. 3–19). Springer US. https://doi.org/10.1007/978-1-4613-8666-7_1

Neumann, D., & Spindler, K. D. (1991). Circasemilunar control of imaginal disc development in Clunio marinus: Temporal switching point, temperature-compensated developmental time and ecdysteroid profile. Journal of Insect Physiology, 37(2), 101-109. https://doi.org/10.1016/0022-1910(91)90095-H

Supplementary Methods: Adjustments in the sampling of adults

Depending on adult density, we either searched the water surface visually to capture individuals with a sieve, or used the sieve to sweep the water surface close to algae. The latter approach yields several individuals per sweep when adults are abundant.

We also adapted our sampling as the season progressed. Initially, we entered the field according to emergence times extrapolated from the known laboratory strains (Kaiser et al. 2021, Briševac et al. 2023), but since real-world emergence times deviated from expectations, we expanded sampling to start as soon as the water receded sufficiently for us to walk to the sites.

Our sampling procedure only records the abundance of swarming adults. However, as adult life span is only 1-2 hours, adult abundance is a relatively good approximation of the temporal distribution of adult emergence from the pupae.

References

Briševac, D., Peralta, C. M., & Kaiser, T. S. (2023). An oligogenic architecture underlying ecological and reproductive divergence in sympatric populations. Elife, 12, e82825. https://doi.org/10.7554/eLife.82825

Kaiser, T. S., von Haeseler, A., Tessmar-Raible, K., & Heckel, D. G. (2021). Timing strains of the marine insect *Clunio marinus* diverged and persist with gene flow. *Molecular Ecology*, *30*(5), Article 5. https://doi.org/10.1111/mec.15791

Supplementary Methods: Multi-locus genotyping

Table S1. Primers for PCR amplification

Locus	Chr	start	end	Forward Primer Sequence	Reverse Primer Sequence
Multiplex 1					
2	1	11755069	11755458	TTAATAACACCTGCTTTGCG	AACGAGTTTAATTAGAAATTTAAAC
3	1	14034017	14034145	GTAGGTAGTTGAAGGTAAATTCTA	GAAATGCTCGACCACATCT
4	1	17280400	17280648	TGGAAAGGAAGCGGTAAAGA	AACAAACTCCCACGCAAAAA
5	1	25531981	25532483	TACTCATGAGGTGTTTCATTAAATTT	TGTGCATATATGTATGCTTTAACATC
8	2	17316253	17316917	TGTTGCAATTTGCTCACG	CGAGATTGCTGTAAGTTATTTTTA
11	3	18085772	18086082	AAAAAAAGAAGGTTCATATCTAAAT	CTGTAGGTTGCCTAGGAG
Multiplex 2					
1	1	8454348	8454703	AACAGGTTTTTGTTCGGTGA	CCAACATCTGAGCCAGCTCTC
6	2	7465168	7465728	GGAAGCCAATCAGTGGAAACC	CCACATTTTCCCATCAATTCG
7	2	9578462	9578670	GTGGTCGTCCGCCTTTAGAT	TACTTGATGACGCGGTGGTT
9	3	10304898	10305352	AGGCTCGTCCGTGACGTATT	CCTTCGACACATTCCTCTTCG
10	3	11136148	11136435	TTTTTGGTTGGGGAGCTTCT	CTGATTCTGATGCACAATTGGA
Insertion-deletion (indel) in the *period* locus					
per	1	17280546	17281110	ACAACGTGACCTGTGACAAT	GAATACTGAGTGTAAGACTTGGC

Genotyping the period locus

Genotyping based on SNPs failed for the *period* locus, the most divergent locus between the FM and NM strains (Briševac et al., 2023). This locus harbors an insertion-deletion (INDEL), making PCR amplification and sequencing inconsistent. We resolved this using PCR primers designed to amplify across the INDEL (Table S1 above; also published in Briševac et al., 2023). In a panel of 12 reference individuals of known strains, the INDEL was present as two alleles: a ~600 bp fragment for the FM strain and a ~1200 bp fragment for the NM strain. The primers were then used to amplify the *period* locus via PCR for all 94 larvae, with the genotype scored in an agarose gel. An individual with one band at ~600 bp was given a score of 1; an individual with one band of ~1200 bp was given a score of 0; an individual with two bands was scored as 0.5. These values entered the multi-locus genotype score in the same way as the scored SNPs.

Reference

Briševac, D., Peralta, C. M., & Kaiser, T. S. (2023). An oligogenic architecture underlying ecological and reproductive divergence in sympatric populations. Elife, 12, e82825. https://doi.org/10.7554/eLife.82825

Supplementary Methods: Modelling details

Table S2. List of variables and parameters.

Name	Explanation	Value
α_z	zone-specific strength of density dependence	see figure legends; constant across all z if the beach has the same steepness profile, variable if steepness varies
$B(v,q)$	Beta distribution	Definition in equation S2
$\beta(d)$	proportion of individuals emerging on day d of the emergence window	defined through the Beta distribution (equation S2)
C	clutch size	50 female offspring per clutch
d	day within an emergence window	1 to 9
$H(t)$	amount of habitat exposed at low tide on day t	varies between 1 and 6, see Figure 1A
I	subscript used to denote the invader strain	invaders initialized at 1% of the population
m	migration towards the water edge	examples use 0.2, 0.5, and 1
$N_{Lz}(t)$	total number of larvae in zone z at time t	outcome of model
$N_{Az}(t)$	total number of emerged adults in zone z at time t	outcome of model
r	daily probability to progress in larval development	0.45, to yield realistic larval development durations (for resulting distribution see Figure S1)
$s_z(t)$	daily survival in zone z	computed based on eq. (1)
s_{max}	daily survival under ideal conditions (unlimited resources)	0.98
$P(d)$	probability that an individual ready to emerge and that has not emerged yet will emerge on day d	derived as a conditional probability based on the Beta distribution (equation S3)

S	strain	takes the value R or I
SC	spatial segregation coefficient	outcome of model (equation S1)
t	time (in days)	proceeds from 1 to 28 within a lunar month
v, q	shape parameters of the Beta distribution (see equation S2)	2 or 5 in the examples
z	zone	1 to 6 (see Figure 1A)

Spatial segregation coefficient

Bonenfant et al. (2007) present an index for sexual segregation that we adopt, interpreting it in a spatial context. Its daily value for our purposes is computed as

$$\mathrm{SC}(t) = 1 - \frac{\sum_{z=1}^{6}(N_{\mathrm{LR}z}(t)+N_{\mathrm{LI}z}(t))}{\sum_{z=1}^{6} N_{\mathrm{LR}z}(t)\cdot\sum_{z=1}^{6} N_{\mathrm{LI}z}(t)} \cdot \sum_{z=1}^{6} \frac{N_{\mathrm{LR}z}(t)N_{\mathrm{LI}z}(t)}{N_{\mathrm{R}z}(t)+N_{\mathrm{L}z}(t)} \tag{S1}$$

where the subscript LIz and LRz denote larvae of the invader and resident strain, respectively, in zone z.

Thereafter we define overall SC as the average over an entire 28-day period.

Emergence window

We model each lunar cycle to offer an emergence window of 9 consecutive days for a given strain.

We use the stretched beta distribution to divide the individuals onto different days within the emergence window:

$$\beta(d) = \left(\frac{1}{9B(v,q)}\right)\left(\frac{d}{9}\right)^{v-1}\left(\frac{9-d}{9}\right)^{q-1} \tag{S2}$$

Here $d = 1\ldots9$ gives the day within the emergence period. For a strain that peaks at day D, $d =$ 1 corresponds to D–4. and $B(v,q)$ is the beta function $B(v,q) = \int_0^1 x^{v-1}(1-x)^{q-1}dx$ with shape parameters v and q. We consider symmetrical parametrizations only that contain a peak in the middle of the emergence period ($v = q > 1$). For a strain that peaks on day d_S, day d=1

of the window is calibrated to occur on day $t = d_S-4$, making the fifth day of emergence the strongest one. The proportion of remaining individuals in the waiting stage that emerge at day d is

$$P(d) = \frac{\int_{d-1}^{d} \beta(t)\,dt}{1 - \int_{0}^{d-1} \beta(t)\,dt} \tag{S3}$$

The value of $P(d)$, multiplied by final-stage larvae in the appropriate zone and strain, yields the number of adult females of the focal strain emerging in zone z on day t.

Equations for oviposition

Table S3. The number of females of strain S ovipositing in each zone, assuming that N_{ASz} of them emerging in zone z at a time when habitat up to $H(t)$ is exposed. The zone-specific number of eggs laid is then 50 times the entry in the table below, as we assume 50 eggs laid per female.

z \ $H(t)$	1	2	3	4	5	6
1	$\sum_{z=1}^{6} N_{ASz}$	$(1-m)N_{AS1}$	$(1-m)N_{AS1}$	$(1-m)N_{AS1}$	$(1-m)N_{AS1}$	$(1-m)N_{AS1}$
2	0	$mN_{AS1} + \sum_{z=2}^{6} N_{ASz}$	$mN_{AS1} + (1-m)N_{AS2}$	$(1-m)N_{AS2}$	$(1-m)N_{AS2}$	$(1-m)N_{AS2}$
3	0	0	$mN_{AS2} + \sum_{z=3}^{6} N_{ASz}$	$mN_{AS2} + (1-m)N_{AS3}$	$(1-m)N_{AS3}$	$(1-m)N_{AS3}$
4	0	0	0	$mN_{AS3} + \sum_{z=4}^{6} N_{ASz}$	$mN_{AS3} + (1-m)N_{AS4}$	$(1-m)N_{AS4}$
5	0	0	0	0	$mN_{AS4} + \sum_{z=5}^{6} N_{ASz}$	$mN_{AS4} + (1-m)N_{AS5}$
6	0	0	0	0	0	$mN_{AS5} + N_{AS6}$

Supplementary figures

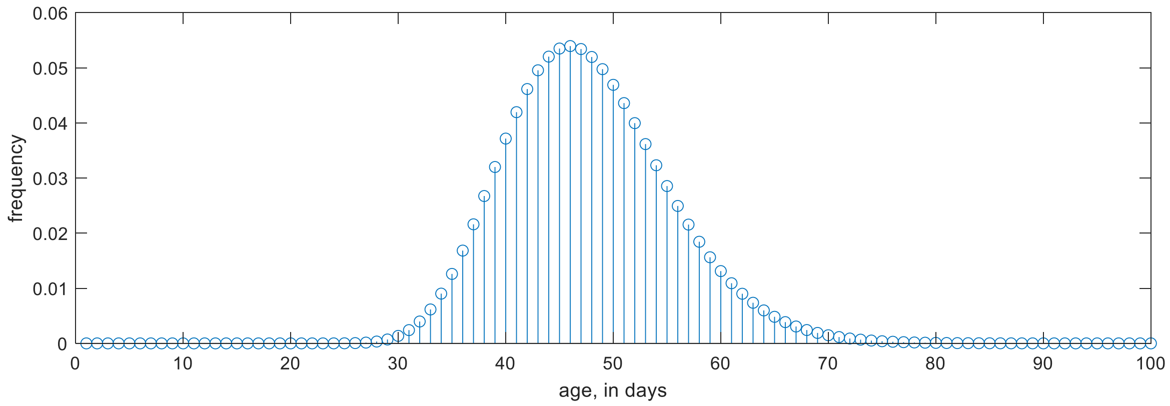

Figure S1. The larval development time distribution (days it takes to complete development) that results from assuming that all larvae survive to complete development, and each experiences a daily probability $r = 0.45$ to move one developmental step further until 21 steps are completed (the complementary probability $1–r$ keeps the larva's developmental stage unchanged for one day). While this distribution indicates the spread of possible developmental durations, the complete model also includes mortality, which depletes the frequency of the longest durations relative to the distribution depicted here. Details of the depletion depend on population density (since we assume mortality is density-dependent). Note that after development, our model still includes a time when the larva waits for the appropriate low tide, thus generation time is longer than the developmental duration time.

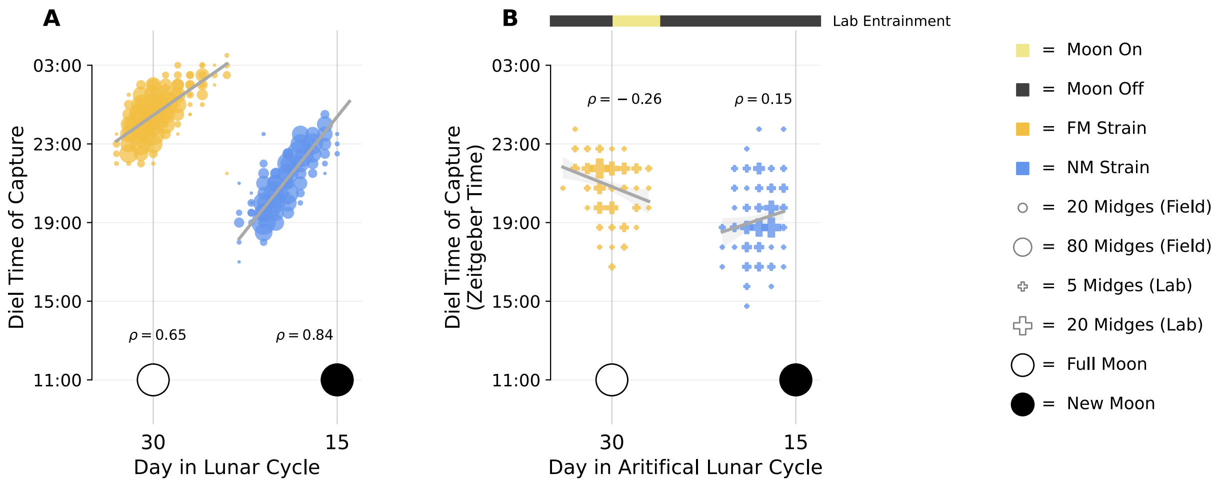

Figure S2. Comparing emergence of Roscoff FM and NM strains (**A**) in the wild versus (**B**) in the laboratory. Laboratory data was recorded for several days in 1-hour intervals with the help of a custom-made fraction collector similar to Honegger (1977). Culture conditions were 20°C, an artificial light-dark cycle of 16:8 hours and artificial moonlight for 4 nights every 30 days. Emergence has been plotted according to the day of the lunar cycle and time that individuals (males) were captured. The full and new moon are indicated by white or black circles. Additionally, in B, the artificial moonlight entrainment schedule is plotted at the top. Grey lines represent lines of best fit, and the Pearson's correlation coefficient (ρ) between day and time of emergence is given for each emergence peak. In the wild, there is a highly significant positive correlation between day and time of emergence (FM strain: p=0.0; NM strain: p=0.0), whereas in the laboratory there is a weak negative correlation (FM strain: p=0.005) or no significant correlation (NM strain: p=0.066). The interpretation of this difference in emergence patterns is that in the laboratory, where there are no tides, daily emergence is only under the control of the circadian clock, which leads to close to constant emergence times every day. In the field emergence is additionally modulated by tidal factors, which ensures that emergence tracks the daily shift in the timing of low tide. In other words, the circadian clock only defines a daily window for emergence, within which emergence is then directly triggered by tidal factors. The laboratory data yield no evidence for a circatidal clock in *C. marinus*.

Reference

Honegger, H.W. 1977. An automatic device for the investigation of the rhythmic emergence pattern of Clunio marinus. International Journal of Chronobiology 4:217-221.

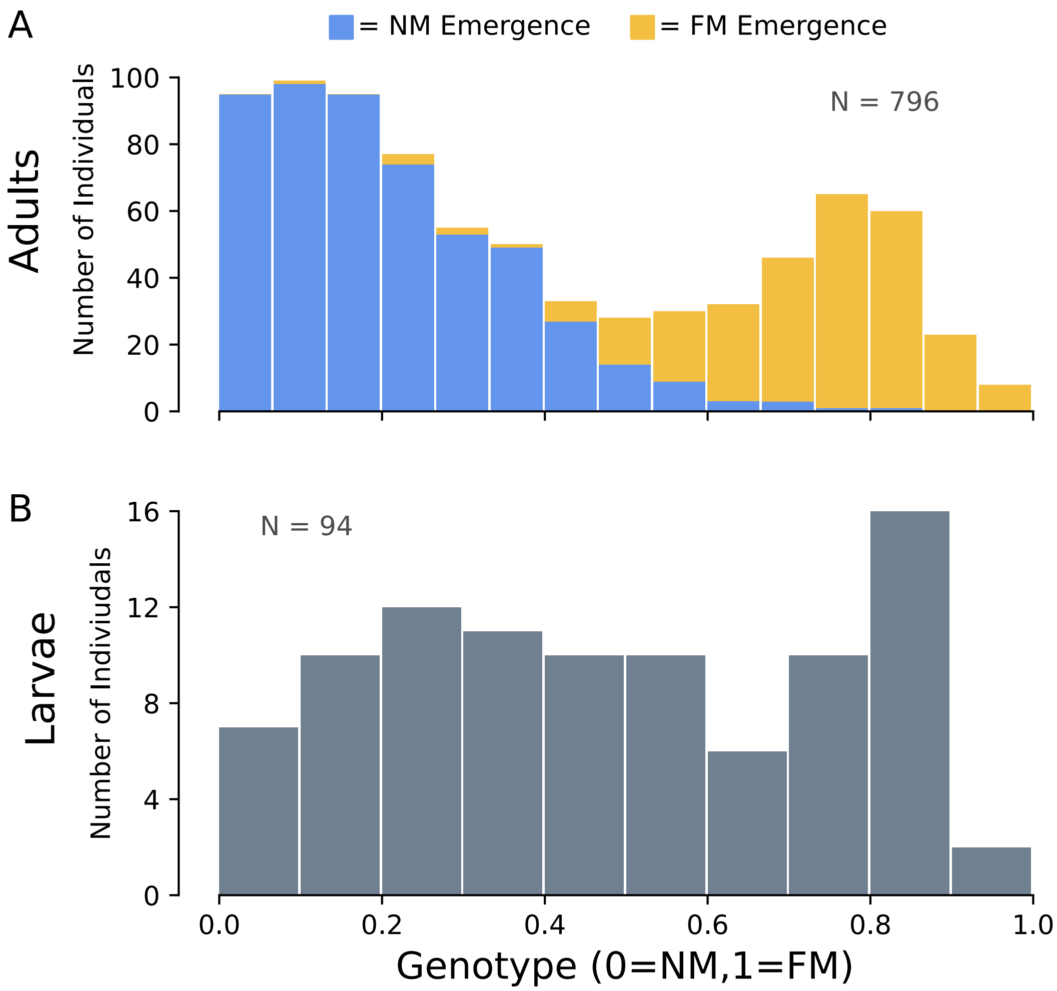

Figure S3 Testing the multi-locus genotyping assay based on field-caught adults which emerged during full moon or new moon respectively. (**A**) Adults caught during new moon have genotype scores closer to 0, whereas adults caught during full moon have genotype scores closer to 1. There is a clear bimodal distribution, with some overlap at intermediate genotype scores. There are very few individuals for which genotype and phenotype do not match. (**B**) The genotypes of the larvae from main Figure 3 recapitulate the bimodal distribution observed in the adults. In the larvae there seems to be a higher incidence of intermediate genotypes, which might suggest that not all of these survive until adult emergence.

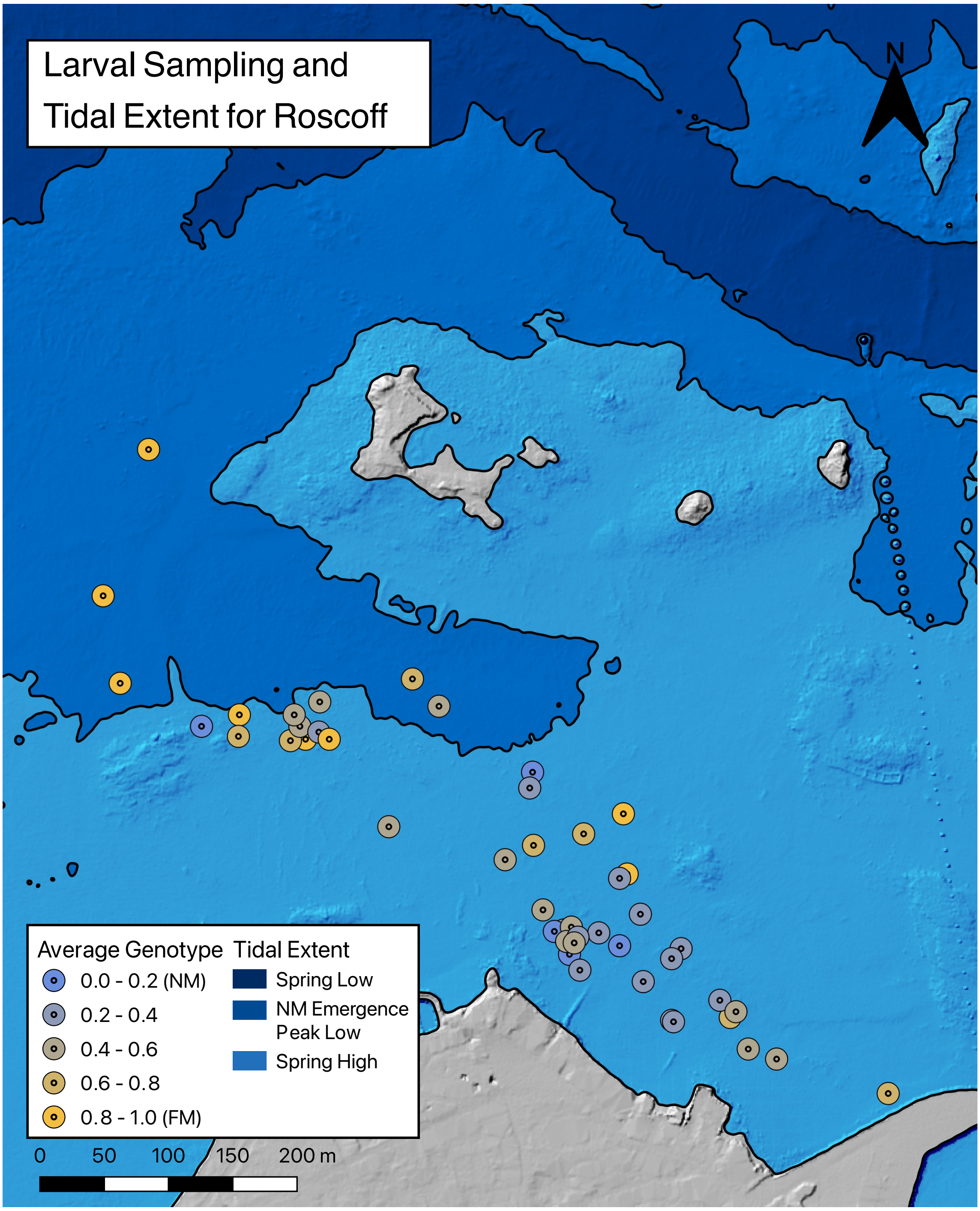

Figure S4. Larval sampling locations in Roscoff, France. Sampling sites have been colored to correspond to the average genotype score at each site, with blue sites having more NM strain individuals, and orange sites having more FM strain individuals. The different color bands represent the water's extent during the average spring low tide, the low tide that occurs during the peak of NM emergence, and the average spring high tide of March-May of 2019.

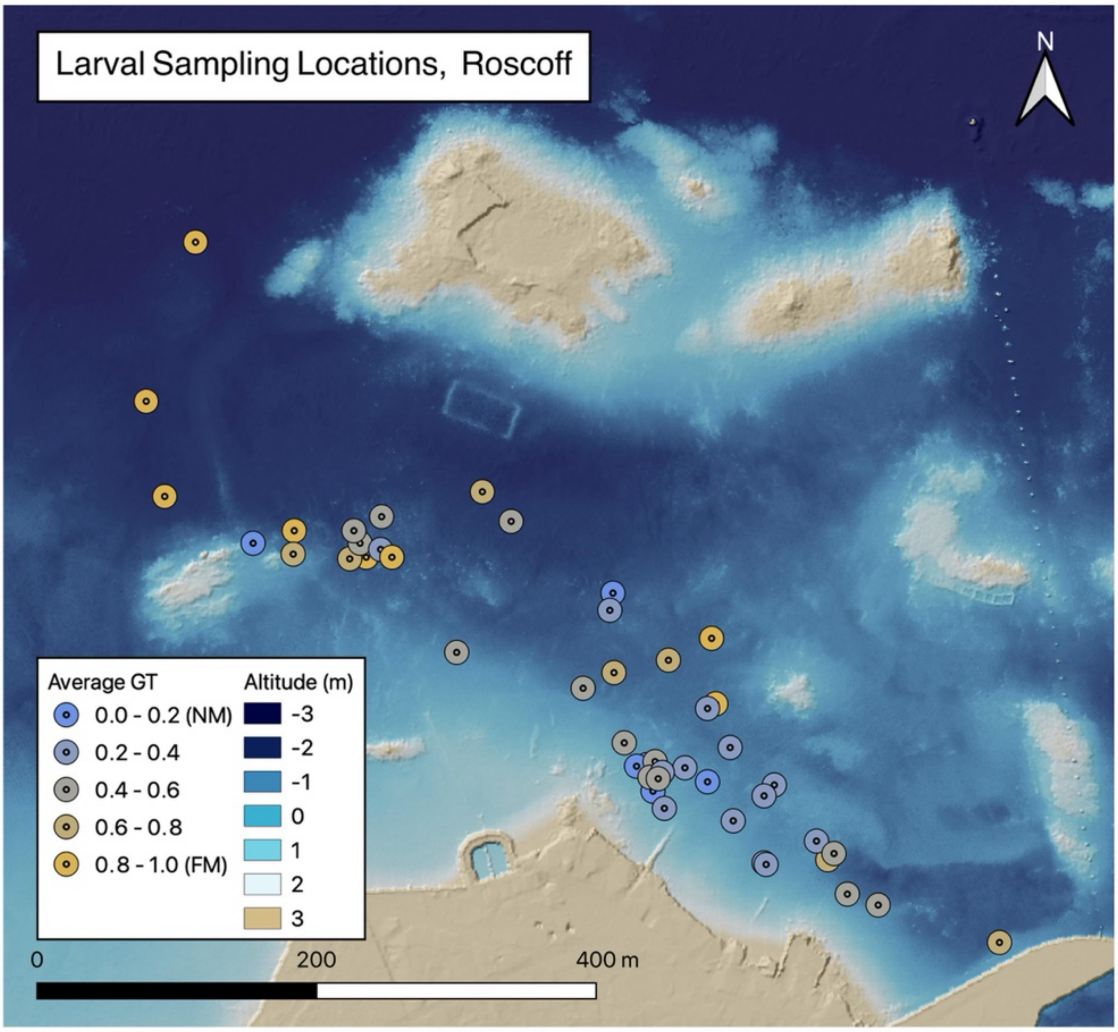

Figure S5. Larval sampling locations in Roscoff, France, colored by the average genotype score for individuals sampled at each site. The base map is shaded to indicate bathymetry (in meters below mean sea level).

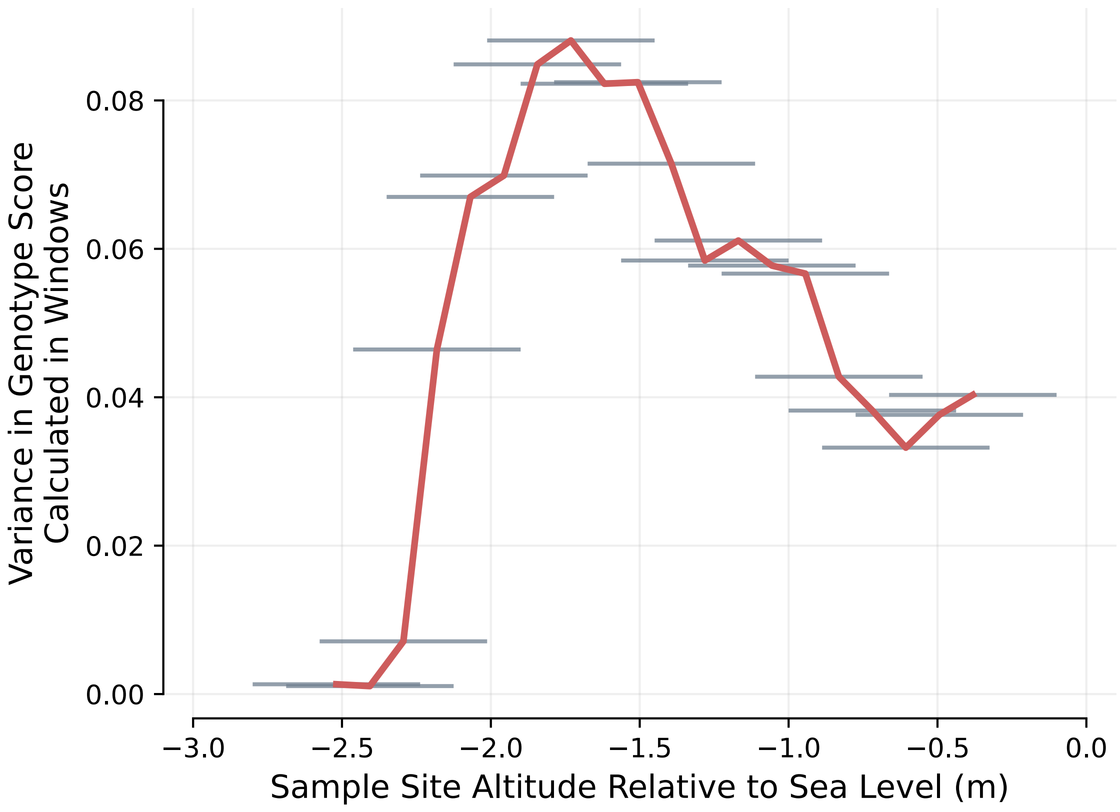

Figure S6. Variation in genotype scores for larvae relative to depth in the intertidal. Variation is highest towards the middle of the intertidal zone, intermediate at higher elevations and low at lower elevations. Variation is calculated in sliding windows, plotted as horizontal grey lines. The red line connects the midpoint of each window.

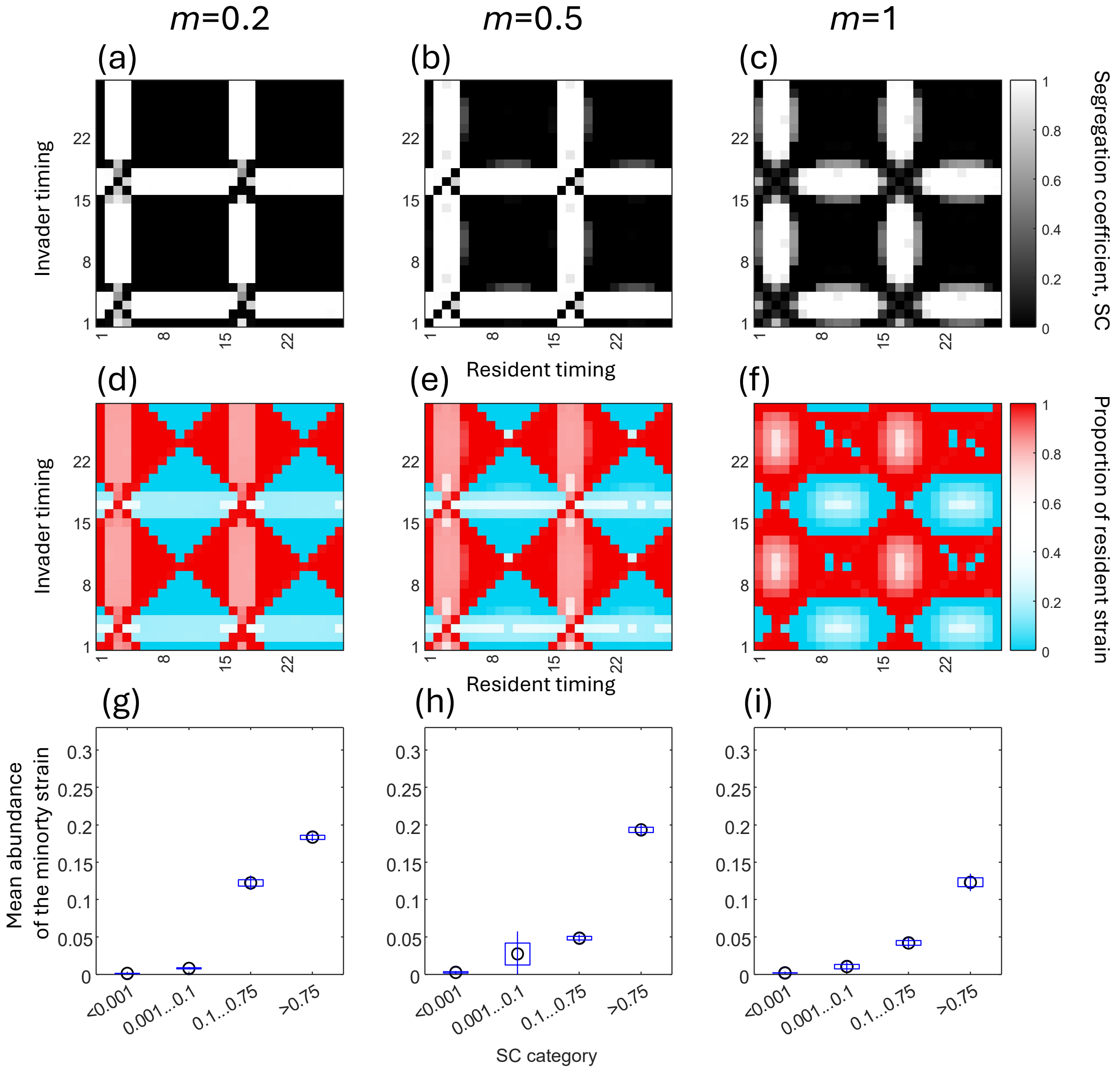

Figure S7. Like Figure 4, but with a pointier distribution of the emergence times, achieved by setting $v = q = 5$.

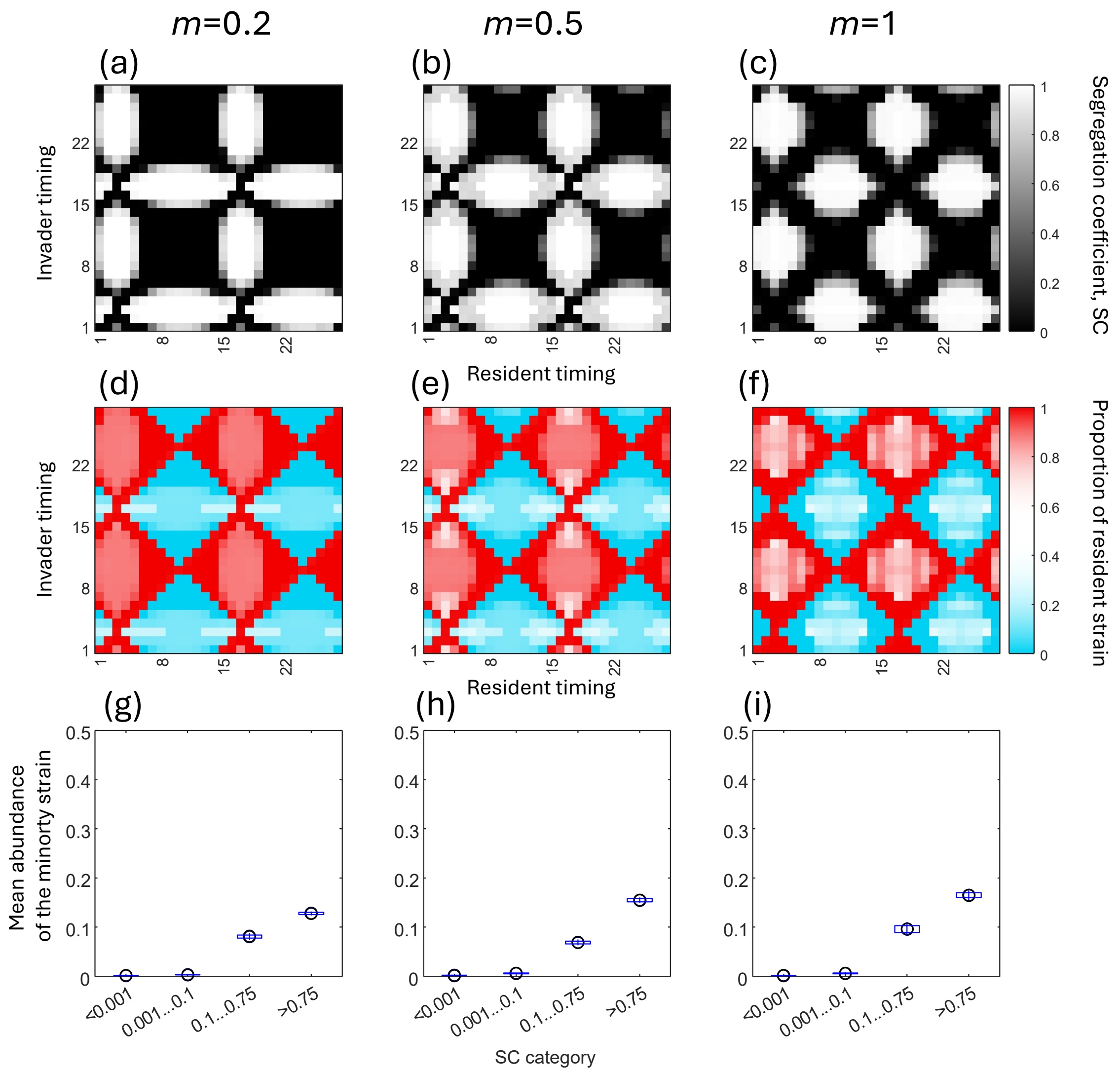

Figure S8. Like Figure 4, but with weaker density dependence in deeper zones (reflecting, e.g., a shallower beach profile as depth increases): $\alpha_1 = 6 \times 10^{-8}$, $\alpha_2 = 5 \times 10^{-8}$, $\alpha_3 = 4 \times 10^{-8}$, $\alpha_4 = 3 \times 10^{-8}$, $\alpha_5 = 2 \times 10^{-8}$, $\alpha_6 = 10^{-8}$.

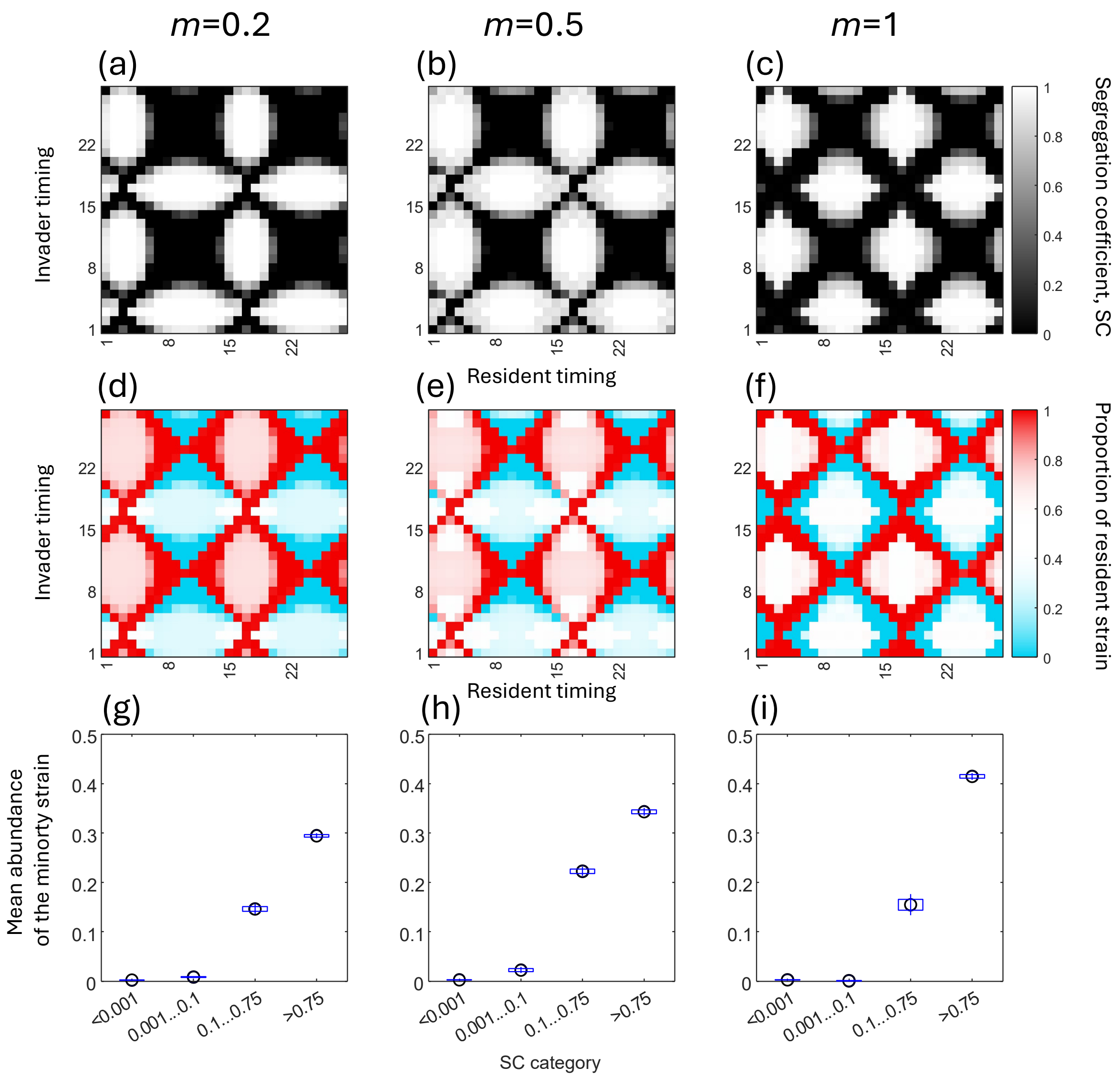

Figure S9. Like Figure S7, but with stronger density dependence in deeper zones (reflecting, e.g., a steeper beach profile as depth increases): $\alpha_1 = 1 \times 10^{-8}$, $\alpha_2 = 2 \times 10^{-8}$, $\alpha_3 = 3 \times 10^{-8}$, $\alpha_4 = 4 \times 10^{-8}$, $\alpha_5 = 5 \times 10^{-8}$, $\alpha_6 = 6 \times 10^{-8}$.
